# Outcomes of highly active antiretroviral therapy and its predictors: a cohort study focusing on tuberculosis co-infection in South West Ethiopia

**DOI:** 10.1186/s13104-015-1417-0

**Published:** 2015-09-15

**Authors:** Jimma Likisa Lenjisa, Sultan Suleman Wega, Tefera Belachew Lema, Gemeda Abebe Ayana

**Affiliations:** Pharmacy Department, College of Medicine and Health Sciences, Ambo University, Ambo, Ethiopia; Pharmacy Department, College of Public Health and Medical Sciences, Jimma University, Jimma, Ethiopia; Population and Family Health Department, College of Public Health and Medical Sciences, Jimma University, Jimma, Ethiopia; Medical Laboratory and Pathology Department, College of Public Health and Medical Sciences, Jimma University, Jimma, Ethiopia

**Keywords:** Outcomes of HAART, TB co-infection, ART, South West Ethiopia

## Abstract

**Background:**

In this study, we hypothesized that TB co-infection independently increases the risk of poor treatment outcomes in such patients even if they are on antiretroviral therapy (ART). Therefore, this study was aimed at investigating this hypothesis among cohort of adult PLWHs in South West Ethiopia.

**Methodology:**

Cohort study comparing the immunologic and clinical outcomes of 130 HIV/TB co-infected and 520 only HIV patients starting ART was enrolled. Chi square and student t test were used to compare outcome variables and logistic regression was used to assess the effect of TB on treatment failure.

**Results:**

In this study, TB co-infection didn’t increase immunologic failure even in univariate analysis at both 6 [OR, 1.10 (0.59–1.69), P = 0.85] and 12 months [OR, 1.06 (0.58–1.93), P = 0.89] of ART initiation. However, it increased the risk of clinical failure at both 6 [Adjusted Odd Ratio (AOR), 2.90 (1.41–6.09), P = 0.028] and 12 months [AOR, 2.93 (1.41–6.09), P = 0.004] of ART initiation.

**Conclusion:**

This study showed that TB co-infection didn’t adversely affect the immunologic outcomes, weight and hemoglobin responses even though it increased the risk of clinical failure nearly three times. Therefore, beside the concern given for TB prevention and treatment, several patient and policy related factors need to be addressed to maximally benefit from highly active antiretroviral therapy rollout in resource limited settings.

## Background

Human immunodeficiency virus (HIV) infection suppresses immunity and increases the risk of opportunistic infections in which tuberculosis (TB) the leading one [1]. There will be about 50 % lifetime risk of developing active TB if an individual is HIV positive [2]. Around 82 % of TB infected people living with HIV (PLWHs) live in sub-Saharan Africa [3]. In Ethiopia the proportion of HIV/TB co infection in the general population is significantly high. Accordingly, it had been reported that most of tuberculosis patients were co-infected with HIV with recent estimates ranging from 46 to 65 % [4].

According to the WHO Global TB Report 2009 (used as background document for the development of this study protocol), Ethiopia ranked seventh in the world for TB burden and third in Africa in 2008, with an estimated TB incidence (all forms) of 378 new cases per 100,000 persons, 163 new smear positive cases per 100,000 persons, and a prevalence (all forms) of 579 per 100,000 population. Following an update to estimates for TB cases and deaths in the African Region, the most recent WHO estimates for Ethiopia are: annual TB incidence (including HIV positive) of 261 per 100,000; prevalence (including HIV positive) of 394 per 100,000 and mortality (excluding HIV) of 35 per 100,000 people [3].

Antiretroviral therapy (ART) remains one of the best strategies in reducing the double trouble imposed by HIV/TB co-infection. However, there are many complications as a result of this co-infection including but not limited to combined adverse drug reactions, pill burden, drug–drug interactions and high non-adherence rate [5–7]. The cumulative effects of these complications could ultimately lead to poor virological, immunological and clinical treatment outcomes in these patient groups [7–9].

However, there are limited literatures which compared outcomes of ART in adult people living with HIV who are TB co-infected with that of PLWH without TB co-infection. Amongst those few literatures available, majority of them are from developed countries [10–12] and published works on this issue in resource poor setting like Ethiopia is scarce [13–15].

In addition to this, the available evidences are not consistent regarding the differences in outcomes of between these two groups of patients when initiated ART. Therefore, in this study, the hypothesis that says TB co-infection independently increases the risk of treatment failure in PLWHs even if they are on ART and fully adherent to such treatment was investigated.

## Methods

### Study area and period

This study was conducted from December to August 2012 in Jimma University Specialized Hospital at ART clinic, South West Ethiopia. Currently the clinic is serving a total of 9809 (3519 on ART and 6290 pre-ART) PLWHs including those with TB co-infection.

In this clinic, patients have regular follow-up for medication refill, and to begin ART when the patients meet the treatment guideline requirement [16]. CD4 determination is at first enrollment, during pre-ART, at the time of ART initiation and every 6 months as per guideline [16].

### Study design and population

A retrospective cohort study was conducted using ART patient master cards and ART patient registers and database. All PLWHs who were on ART at this clinic and started such treatment between January 2008 and March 2011 were our population. A total of 260 and 1100 patients with and without TB were enrolled respectively during these periods fulfilling the inclusion criteria. Of these, we included only adult PLWH (age ≥15 years) who were on ART and had at least one follow up clinic visit after initiation of ART using simple random sampling. In this case, the source population had been categorized into two groups and those who fulfilled the inclusion criteria were listed according to their registration number. This made the sampling frame for both groups from which the required sample size was taken using simple random sampling (every other patients record from the list). Standard table of sample size for cohort studies was used to determine sample size for this study [17].

**Fig. 1:**
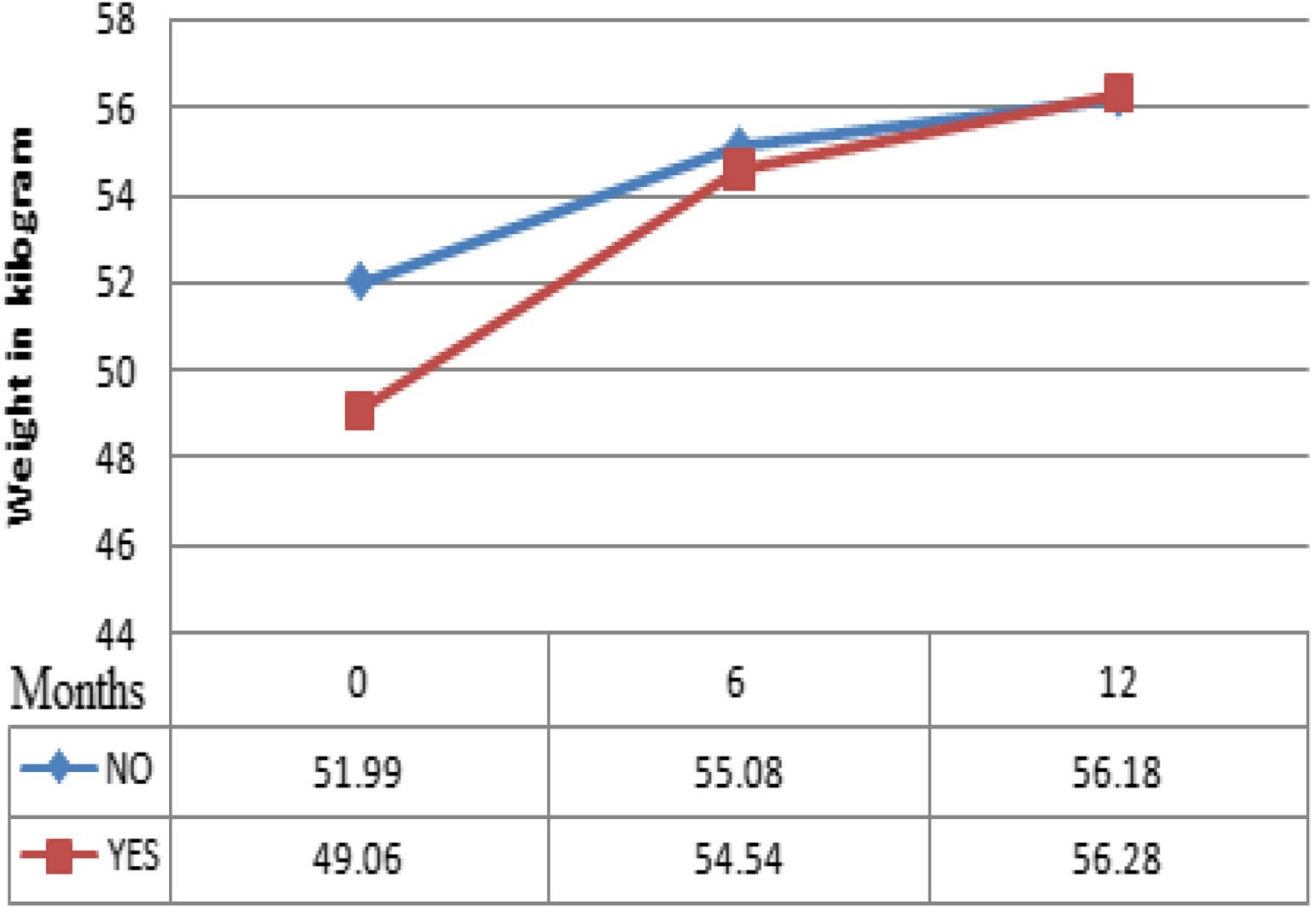
Mean weight measured at baseline (0), 6 and 12 months on ART for PLWHs with TB (*bottom line* designated as ‘YES’) and without TB (*top line* designated as ‘NO’), South West Ethiopia, 2012

Accordingly, the final sample size was determined to be 650 (PLWH with TB co-infection = 130, and PLWH without TB = 520) with 90 % power of the study after considering 16 % for possible incompleteness of the recordings and 10 % for lost to follow up cases. Data collection was done using a pretested data abstraction format that was designed for the purpose of this study. It was undertaken by well trained pharmacists up on the supervision of the principal investigator (Fig. 1).

In this study, immunologic failure has been defined as fall of CD4 count to pre-therapy baseline (or below); or 50 % fall from the on-treatment peak value (if known); or Persistent CD4 levels below 100 cells/mm^3^ [18]. Clinical treatment failure means new or recurrent WHO stage 4 conditions for adults and adolescents. Certain WHO clinical stage 3 conditions (e.g. pulmonary TB, severe bacterial infections), may be an indication of treatment failure [18]. And AIDS defining illnesses in this study include conditions listed in the 1993 Expanded AIDS Surveillance Case Definition for Adolescents and Adults as per center for disease control and prevention (CDC) [19].

### Data analysis

Statistical package for social sciences (SPSS) version 20.0 was used for this purpose. All tests were two-tailed and P < 0.05 considered significant. Chi square (χ^2^) test was used to compare categorical outcome variables. For continuous variables, independent sample student t test was used. Binary logistic regression was performed to assess the effect of TB co-infection on clinical and immunologic failure. Categorical variables were reported as frequency (%), and continuous variables as mean ± std. error mean (standard error of mean).

### Ethical consideration

Ethical clearance was obtained from the ethical review board of Jimma University College of Public Health and Medical Sciences. During data collection, any personally identifiable information was not included in the data collection format except the ART unique number of each patient to keep confidentiality. No need of securing verbal or written consent as it was secondary data.

## Results

### Characteristics of the patients at ART initiation

Analysis was made in comparison of two groups of study cohorts; PLWHs with HIV/TB co-infection (n = 130) and with only HIV (n = 520) at ART initiation. As shown in Table 1, the characteristics of patients were comparable except that there was significant sex difference (p = 0.017). The mean age was 33.6 ± 0.4 and 33.4 ± 0.8 years respectively for PLWHs without and with TB co-infection. The mean values of weight, CD4 cell count and BMI at ART initiation were all significantly higher for PLWHs with only HIV infection. Both groups of patients were most commonly prescribed non TDF based regimens which accounted for 325 (63.3 %) and 76 (59.2) of PLWHs without and with TB co-infection respectively.

**Table 1:** Characteristics of the Patients at ART initiation, South West Ethiopia, 2012

Variables	Category	TB status		p value
		No (N = 520)	Yes (N = 130)	
Age in year, mean ± std. error of mean		33.6 ± 0.4	33.4 ± 0.8	0.850
Gender	Male, N (%)	165 (31.7)	56 (43.1)	0.017
	Female, N (%)	355 (68.3)	74 (56.9)	
Residence	Rural, N (%)	125 (24)	35 (26.9)	0.569
	Urban, N (%)	395 (76)	95 (73.1)	
Marital status	Never married N (%)	102 (19.6)	26 (20.0)	0.160
	Ever married, N (%)	418 (80.4)	104 (80.0)	
Educational level	Illiterate, N (%)	102 (19.6)	37 (28.5)	0.168
	Primary, N (%)	183 (35.2)	39 (30)	
	Secondary, N (%)	175 (33.7)	39 (30)	
	Tertiary, N (%)	60 (11.5)	15 (11.5)	
Employment status	Unemployed, N (%)	350 (67.3)	91 (70.0)	0.257
	Employed, N (%)	170 (33.7)	39 (30.0)	
Drug addiction behavior	No, N (%)	246 (47.3)	57 (43.8)	0.479
	Yes, N (%)	274 (52.7)	73 (56.2)	
Weight in kg, mean ± std. error of mean		52.0 ± 0.4	49.1 ± 0.8	0.003
CD4 in cells/mm^3^, mean ± std. error of mean		153.8 ± 3.8	128.7 ± 9.7	0.006
BMI in kg/m^2^, mean ± std. error of mean		19.4 ± 0.1	18.2 ± 0.3	<0.001
Hgb mg/dl, mean ± std. error of mean		12.3 ± 0.1	11.03 ± 0.2	<0.001
WHO clinical stage	Stage I/II, N (%)	334 (64.2)	0 (0)	<0.001
	Stage III, N (%)	157 (30.2)	85 (65.4)	
	Stage IV, N (%)	29 (5.6)	45 (34.6)	
ART regimen	Non TDF based, N (%)	325 (63.3)	76 (59.2)	<0.001
	TDF-based, N (%)	196 (37.7)	53 (40.8)	
Functional status	Working, N (%)	378 (72.7)	51 (38.7)	<0.001
	Ambulatory, N (%)	127 (24.4)	66 (50.8)	
	Bed ridden, N (%)	15 (2.9)	13 (10)	

### Immunologic failure

In this study, crude analysis indicated that immunologic failure was significantly higher in HIV only infected patients; 141 (27.1 %) as compared to those with TB co-infection 29 (22.3 %) at 6 months after initiation of ART considering on treatment analysis (p = 0.043). Similar trend had been obtained at 12 months of follow up with a failure proportion of 102 (19.6 %) in PLWHs without TB versus 18 (13.8 %) in those with TB co-infection (p < 0.001). See Table 2.

**Table 2:** Outcomes of ART compared based on TB status, South West Ethiopia, 2012

ART outcomes	Months	TB status		p value
		No (N = 520)	Yes (N = 130)	
Clinical failure, N (%)	6	95 (18.3)	46 (35.4)	<0.001**
	12	62 (11.9)	38 (29.2)	<0.001**
Immunologic failure, N (%)	6	141 (27.1)	29 (22.3)	<0.001**
	12	102 (19.6)	18 (13.8)	0.043
AIDS defining illness, N (%)	6	27 (5.2)	14 (10.8)	0.005**
	12	20 (3.8)	11 (8.5)	<0.001
CD4 cells increase, mean ± std. error of mean	6	146.7 ± 7.2	150.5 ± 14.1	0.827*
	12	180.7 ± 8.1	186.1 ± 17.6	0.780
Hemoglobin increase, mean ± std. error of mean	6	1.0 ± 0.1	1.8 ± 0.4	0.028*
	12	1.3 ± 0.1	2.0 ± 0.3	0.046
Weight gain in kg, mean ± std. error of mean	6	2.8 ± 0.2	4.4 ± 0.6	0.008*
	12	3.9 ± 0.3	5.9 ± 0.8	0.023

** Compared using Chi square test, * compared using independent sample student t test

**Fig. 2:**
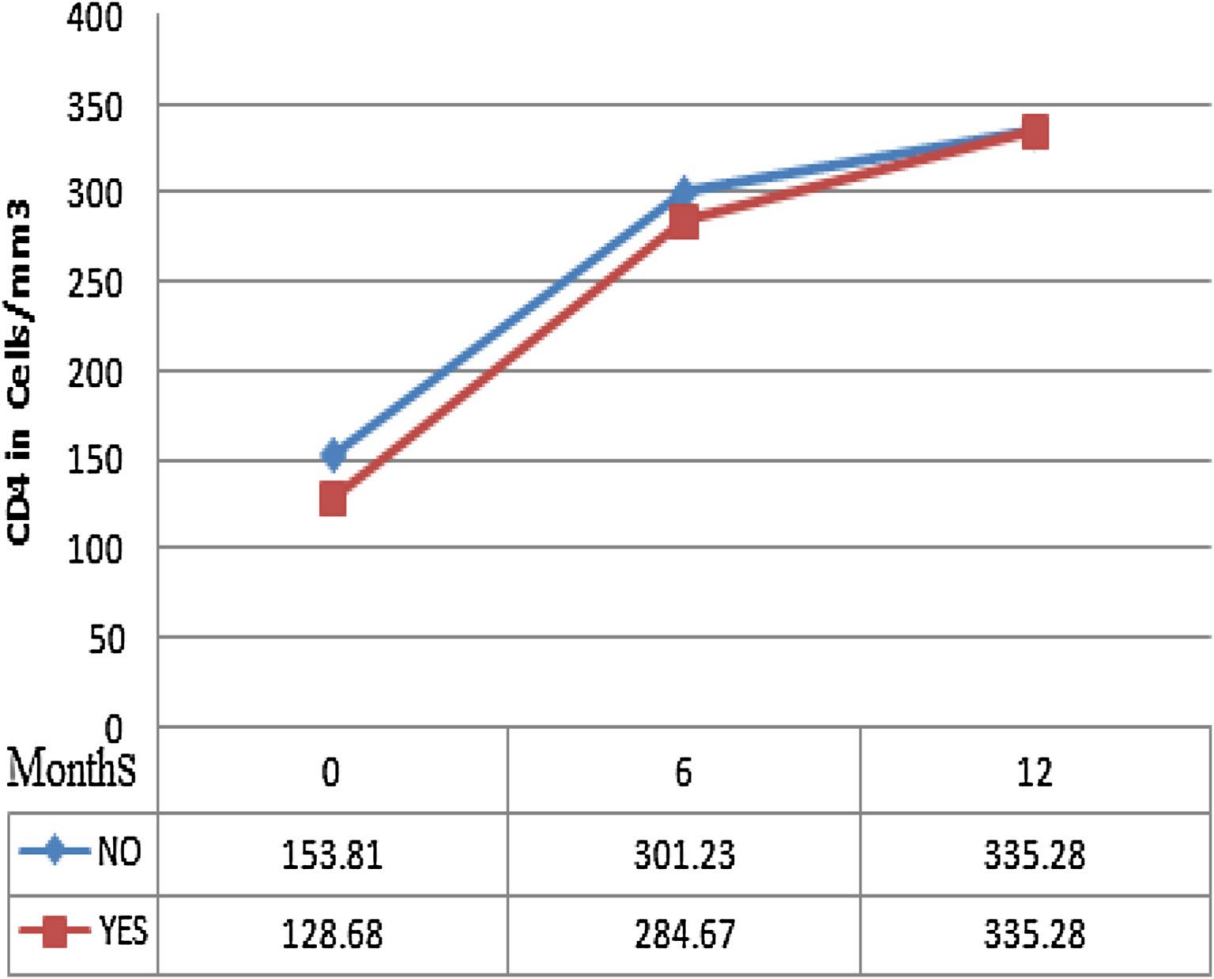
Mean CD4 cells count measured at baseline (0), 6 and 12 months on ART for cohorts with TB (*bottom line* designated as ‘YES’) and without TB (*top line* designated as ‘NO’), South West Ethiopia, 2012

**Fig. 3:**
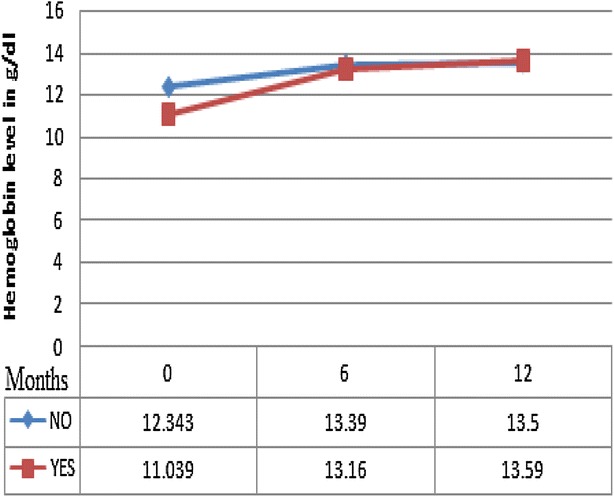
Mean hemoglobin level measured at baseline (0), 6 and 12 months on ART for cohorts with TB (*bottom line* designated as ‘YES’) and without TB (*top line* designated as ‘NO’), South West Ethiopia, 2012

Table 3 shows the result of logistic regression analysis in assessing the effect of tuberculosis co-infection on immunologic failure rate at 6 and 12 months of follow up periods. Accordingly, the presence of TB co-infection had been shown to have no significant effect on immunologic failure even in univariate analysis at both six [OR, 1.10 (0.59–1.69), p < 0.85] and twelve [OR 1.06 (0.58–1.93), p = 0.89] months of follow up periods. Rather, the effect of other factors like baseline CD4 cell counts of <50 cells/mm^3^ [AOR 12.0 (1.26–13.79), p = 0.03], and 101–200 cells/mm^3^ [AOR 9.5 (1.08–14.39), p = 0.04] and low weight [AOR 0.98 (0.954–0.998), p = 0.03] at ART initiation was seen. In this case, for every 1 kg lower in weight at initiation of treatment, patients are 2 % more likely to experience immunologic failure (Figs. 2, 3).

**Table 3:** Logistic regression model for assessing the effect of TB co-infection on immunologic failure, December 2011–August 2012, JUSH, Ethiopia

Logistic regression	Follow up time in months							
	At 6 months of ART initiation				At 12 months of ART initiation			
	Univariate analysis		Multivariate analysis		Univariate analysis		Multivariate analysis	
	OR (95 % CI)	P	AOR (95 % CI)	P	AOR (95 % CI)	P	AOR (95 % CI)	P
Variables								
TB status								
Absent	Reference	–	Reference		Reference	–	Reference	–
Present	1.10 (0.59–1.69)	0.985	0.96 (0.56–3.46)	0.540	1.06 (0.58–1.93)	0.839	1.04 (0.46–1.89)	0.740
Age in years	0.98 (0.96–1.01)	0.100	0.76 (0.75–2.23)	0.062	0.97 (0.95–0.99)	0.012	0.98 (0.95–0.99)	0.049
Weight in kg	0.97 (0.95–0.99)	0.011	0.98 (0.95–0.99)	0.030	0.99 (0.96–1.01)	0.218	0.37 (0.089–3.45)	0.347
CD4 count category in cells/mm^3^								
<50	14.2 (1.52–16.42)	0.020	12.0 (1.26–13.79)	0.031	24.67 (1.25–32.53)	0.005	19.97 (2.09–34.52)	0.009
50–100	10.4 (1.45–14.53)	0.003	9.8 (1.26–12.80)	0.044	13.43 (1.52–18.63)	0.019	12.28 (1.37–14.09)	0.025
101–200	9.6 (1.09–13.87)	0.041	9.50 (1.08–14.39)	0.043	15.52 (1.82–26.48)	0.012	14.39 (1.66–23.49)	0.015
>200	Reference	–	Reference	–	Reference	–	Reference	–

However, the risk of immunologic failure decreases as the age at ART initiation becomes older and older [AOR 0.98 (0.95–0.99), P = 0.04] at 12 months. For this reason, for every 1 year older at initiation of ART, the risk of immunologic failure decreases by 2 %.

### Clinical failure

Crude analysis showed that after 6 months of ART initiation, clinical failure was significantly higher in PLWHs with TB co-infection; 35.4 % compared to those with no TB co-infection; 18.3 % (P < 0.001). Of those who failed clinically, 5.2 % of PLWHs without TB and 10.8 % with TB co-infection developed new AIDS defining illnesses (P = 0.005). Likewise, at 12 months of follow up, clinical failure was higher in PLWHs with TB co-infection by 17.3 % (P < 0.001). At this time, of those who failed clinically, new AIDS defining illnesses were 3.8 % in PLWHs without TB and 8.5 % in those with TB (P < 0.001) as indicated in Table 2.

As Table 4 shows the presence of TB confection at baseline [AOR, 2.90 (1.41–6.09), P = 0.028] increased the risk of clinical failure at 6 months of follow up. Beside tuberculosis, factors such as WHO clinical stage III [AOR 17.83 (7.44–42.73), P < 0.001] and stage IV [AOR 17.06 (5.12–56.80), P < 0.001] and being ambulatory at baseline [AOR 1.86 (1.049–3.286), P = 0.034] were the predictors of clinical failure at 6 months of follow up. Similarly, at 12 months, the presence of TB co-infection [AOR 2.93 (1.412–6.094), P = 0.004] continued to increase the risk of clinical failure. Other baseline variables such as working parttime [AOR, 6.07 (1.63–22.65), P = 0.007], having drug addiction behavior [AOR 1.76 (1.02–3.03), P = 0.04], WHO stage III [AOR 6.98 (3.03–16.08), P < 0.001] and WHO stage IV [AOR 6.88 (2.09–22.64), P = 0.002] were shown to be independent predictors of clinical failure at 12 months of ART initiation in addition to tuberculosis co-infection.

**Table 4:** Logistic regression model for assessing the effect of TB co-infection on clinical failure, December 2011–August 2012, JUSH, Ethiopia

Logistic regression	Follow up time in months							
	At 6 months of follow up visits				At 12 months of follow up visits			
	Univariate analysis		Multivariate analysis		Univariate analysis		Multivariate analysis	
	OR (95 % CI)	P	AOR (95 % CI)	P	AOR (95 % CI)	P	AOR (95 % CI)	P
Variables								
TB status at base line								
Absent	Reference	–	Reference	–	Reference		Reference	
Present	3.0 (1.90–4.64)	0.001	2.90 (1.41–6.09)	0.028	4.62 (2.79–7.64)	<0.001	2.93 (1.41–6.09)	0.004
Employment status at base line								
Work fulltime	Reference	–	Reference	–	Reference	–	Reference	–
Work parttime	4.62 (1.89–11.74)	0.005	5.48 (1.96–15.39)	0.042	5.68 (2.01–16.13)	0.001	6.07 (1.63–22.65)	0.007
Drug addiction behavior at base line								
Absent	Reference	–	Reference		Reference	–	Reference	–
Present	1.24 (0.98–4.55)	0.076	1.20 (0.78–3.65)	0.086	1.71 (1.09–2.67)	0.020	1.76 (1.02–3.03)	0.041
Base line WHO stage								
I/II	Reference	–	Reference	–	Reference	–		
III	16.30 (7.87–33.70)	0.001	17.83 (7.44–42.7)	<0.001	8.72 (4.01–19.03)	<0.001	6.58 (3.03–16.08)	<0.001
IV	16.44 (7.05–38.37)	0.001	17.06 (5.12–56.80)	<0.001	19.17 (7.82–46.97)	<0.001	6.98 (2.09–22.64)	0.002
Baseline functional status								
Working	Reference	–	Reference	–	Reference	–		
Ambulatory	3.74 (2.49–5.62)	0.001	1.86 (1.05–3.28)	0.034	3.58 (2.26–5.67)	0.001	2.56 (3.47–8.98)	0.075

### CD4 cells, hemoglobin and weight responses on antiretroviral regimens

The mean gains in CD4 cells at 6 months of follow up periods was 150.5 ± 14.1 cells/mm^3^ in PLWHs with TB co-infection and 146.7 ± 7.2 cells/mm^3^ in those without TB co-infection. At 12 months it was 180.7 ± 8.1 in PLWHs with TB co-infection and 186.1 ± 17.6 cells/mm^3^ in those without TB co-infection. However, these differences were statistically insignificant at both 6 months (P = 0.83) and 12 months (P = 0.78) of ART initiation.

However, the mean hemoglobin gains were significantly greater in PLWHs with TB co-infection; 1.0 ± 0.1 versus 1.8 ± 0.4 g/dl at 6 months (P = 0.028) and 1.3 ± 0.1 versus 2.0 ± 0.3 g/dl at 12 months (p = 0.046). Similarly, the mean weight gains was significantly greater in PLWHs with TB co-infection; 2.8 ± 0.2 versus 4.4 ± 0.6 kg at 6 months (p = 0.008) and 3.9 ± 0.3 versus 5.9 ± 0.8 kg at 12 months (p = 0.023). See Table 2 for better illustration of these results.

## Discussion

In this study, the difference in outcomes of first line antiretroviral regimens between two groups of patients: PLWHs with and without TB co-infection during the initiation of ART were compared. In addition, the effect of tuberculosis on these outcome variables was assessed and discussed accordingly.

In this study, when the mean increases in CD4 cells count were compared the difference was not significant for both groups of PLWHs in contrary to what had been reported as starting ART, particularly early during TB treatment, could jeopardize adherence to treatment and hence worsening of the immune response [20]. Hung et al. [10], Sharma and colleagues [21], Sinha et al. [12] and Lipman et al. [11] also showed similar findings. According to one study the additional increment in CD4 count in patients with co-infection following treatment suggest that CD4 suppression at the onset of TB may be the direct result of mycobacterium growth and inflammation as well as interaction between TB and HIV in addition to the effect of HIV alone [21]. Hence, up on appropriate treatment for both diseases, greater degree of CD4 cells upsurge can occur. Moreover, a study done in London showed that study participants in the EFV group had a CD4 count increase of on average 26 cells/mm^3^ higher than those in the NVP group [22]. As majority of study participants with TB co-infection in this study were on EFV based regimens, they could recover their immunity faster as a result.

TB co infection at initiation of ART, in this study, was found to have no impact on immunologic failure in both univariate and multivariate analysis. This is similar to a 3 years pediatric ART outcome study in Ethiopia [23] and a 4 years adult ART outcome study in Mozambique [22]. It has been further demonstrated that concurrent TB co-infection had not increased the risk of immunologic failure in South Africa [24].

In the present study, clinical failure and hence HIV progression was also determined and compared between PLWHs with and without TB co-infection. Accordingly, it was observed to be significantly higher in PLWHs with TB co-infection. In Taiwan, similar finding was reported [3]. This could be due to the fact that PLWHs with TB co-infection had additionally greater proportion of other AIDS defining conditions when initiating ART. This had been shown in one study conducted in united state as well [25].

Furthermore, studies are reporting that drug abuse such as alcohol after contracting HIV seems to accelerate disease progression through a direct effect on CD4+ cells and also indirectly by increasing medication non-adherence rate [26, 27]. Therefore, as larger number (but not statistically significant) of such patients found in PLWHs with TB co infection group in this study, it might contribute to the significantly worse HIV progression in the later group of patients. The result of our multivariate logistic regression analysis also confirmed that tuberculosis co-infection increased the risk of clinical failure and also HIV progression several folds.

Studies are claiming that hemoglobin level and weight can be utilized as important tools for monitoring outcomes of antiretroviral therapy in resource limited settings like Ethiopia [28, 29]. We also compared these variables between PLWHs with and without TB co infection who were on treatment in this study.

Accordingly, the difference in mean values of weight gain was significant for the two groups of PLWHs. It was observed that patients without TB, in this study, had greater weights at baseline. Nevertheless, the amount of change with time was significantly higher in HIV/TB co infected patients. This finding was in agreement with that of study conducted in India [21]. This could be due to the fact that in more sick individuals, such parameter has to increase faster during treatment (both anti TB and ART) as they need to attain medically required states faster. It could occur as a result of improvement in nutritional status after initiation of HAART and TB therapy [30]. Moreover, this weight gain is presumed to be caused by the suppression of viral replication in HIV-infected individuals on HAART [31].

The present study also compared the average increase in hemoglobin for the two groups of patients. Studies showed that pulmonary tuberculosis as well as other AIDS defining opportunistic infections are associated with a higher risk of anemia among HIV-positive patients [32, 33]. Regardless of this fact, the increase in hemoglobin on ART in this study was significantly greater for PLWHs with TB confection even if the later has lower hemoglobin level at the initiation of treatment.

### Limitations

Limitations of the present study primarily relate to the fact that analyses were based on routinely collected data, which were incomplete for certain baseline and follow-up clinical characteristics. Another limitation is that there could be misclassification bias as it is not easy to diagnosis TB in HIV positive patients. As a result, HIV/TB co-infected patients may be treated only for HIV and also only HIV infected patients may be treated wrongly for TB as well.

## Conclusion

This study showed that TB co-infection didn’t significantly increase the risk of immunologic failure. Weight and hemoglobin responses were even better in co-infected patients. However, these patients were more likely to experience clinical failure and that the co-infection roughly triples the risk of clinical failure. In general, for better outcomes of ART rollouts in Ethiopia the following points need to be addressed. Primarily, there should be improved strategies to ensure patients present early to health facilities. In addition, earlier nutritional intervention, prevention of TB, establishing dedicated adolescent and youth services at ART clinics and addressing drug addiction behavior of individuals on ART could improve ART outcomes.
